# Cryptic ecology among host generalist *Campylobacter jejuni* in domestic animals

**DOI:** 10.1111/mec.12742

**Published:** 2014-04-25

**Authors:** Samuel K Sheppard, Lu Cheng, Guillaume Méric, Caroline P A de Haan, Ann-Katrin Llarena, Pekka Marttinen, Ana Vidal, Anne Ridley, Felicity Clifton-Hadley, Thomas R Connor, Norval J C Strachan, Ken Forbes, Frances M Colles, Keith A Jolley, Stephen D Bentley, Martin C J Maiden, Marja-Liisa Hänninen, Julian Parkhill, William P Hanage, Jukka Corander

**Affiliations:** * Department of Zoology, University of OxfordThe Tinbergen Building, South Parks Road, Oxford, OX1 3PS, UK; † Institute of Life Science, College of Medicine, Swansea UniversitySwansea, SA2 8PP, UK; ‡ Department of Mathematics and Statistics, University of HelsinkiP.O. Box 68, FI-00014, Helsinki, Finland; § Department of Food Hygiene and Environmental Health, University of HelsinkiP.O. Box 66, FI-00014, Helsinki, Finland; ¶ Department of Information and Computer Science, Helsinki Institute for Information Technology HIIT, Aalto UniversityP.O. Box 15400, FI-00076, Aalto, Finland; ** Department of Bacteriology and Food Safety, Animal Health and Veterinary Laboratories Agency (AHVLA)New Haw, Addlestone, Surrey, KT15 3NB, UK; †† Cardiff School of Biosciences, Cardiff UniversityMain Building, Park Place, Cardiff, CF10 3AT, UK; ‡‡ School of Medicine and Dentistry, University of AberdeenForesterhill, Aberdeen, AB25 2ZD, UK; §§ School of Biological Sciences, University of AberdeenForesterhill, Aberdeen, AB25 2ZD, UK; ¶¶ Wellcome Trust Sanger InstituteWellcome Trust Genome Campus, Hinxton, Cambridge, CB10 1SA, UK; *** Department of Epidemiology, Harvard School of Public HealthKresge Building, 677 Huntington Avenue, Boston, MA, 02115, USA

**Keywords:** adaptation, *Campylobacter*, genomics, recombination barriers

## Abstract

Homologous recombination between bacterial strains is theoretically capable of preventing the separation of daughter clusters, and producing cohesive clouds of genotypes in sequence space. However, numerous barriers to recombination are known. Barriers may be essential such as adaptive incompatibility, or ecological, which is associated with the opportunities for recombination in the natural habitat. *Campylobacter jejuni* is a gut colonizer of numerous animal species and a major human enteric pathogen. We demonstrate that the two major generalist lineages of *C. jejuni* do not show evidence of recombination with each other in nature, despite having a high degree of host niche overlap and recombining extensively with specialist lineages. However, transformation experiments show that the generalist lineages readily recombine with one another in vitro. This suggests ecological rather than essential barriers to recombination, caused by a cryptic niche structure within the hosts.

## Introduction

The degree to which pathogenic bacteria are adapted to specific host niches is a key to understanding their epidemiological and evolutionary dynamics. Expansion into a new host species is often thought to be accompanied by evolutionary specialization and gradual divergence from the ancestral population. However, in practice, many pathogens are not specialized to living in only one species but can colonize multiple hosts suggesting that host generalism can also be a successful ecological strategy (Woolhouse *et al.* 2001). In bacteria such as *Staphylococcus aureus,* where livestock-associated lineages are largely host-restricted, comparing genotype information with ecology can be instructive in determining the direction of host jumps and the timescale of specialization (Lowder *et al.* 2009). However, the relationship between genetic clusters and ecological niche is not clear in many organisms, such as *Escherichia coli* (Meric *et al.* 2012).

The ability of *Campylobacter jejuni* to colonize the gastrointestinal tracts of multiple bird and mammal hosts (often benignly), and infect humans via food and the environment, is well documented (Sheppard *et al.* 2011). Genotyping large numbers of strains from various sources by multilocus sequence typing (MLST), has identified lineage segregation by host, but with substantial overlap in some strains and evidence for ubiquitous lineages that occupy multiple host niches (Sheppard *et al.* 2011). For example, some clonal complexes are strongly associated with either chicken or cattle, while others are commonly found in both of these host species as well as in wild birds (Sheppard *et al.* 2010; Gripp *et al.* 2011). This type of host generalism may seem an unlikely strategy from an evolutionary and adaptive point of view, because of out-competition by host specialists and selection pressures to co-evolve with more than one host. One possible explanation for the coexistence of multiple lineages could be that they occupy different unknown subniches within the same hosts. This leads to questions about how different cryptic ecological strategies evolve. Specifically, are there multiple generalist and specialist lineages? How are they related to one another? Can ecological factors explain generation and maintenance of lineage clusters? Here, we use a combination of population scale genetic analysis of MLST data, comparative genomics, and in vitro recombination experiments to investigate the evolution of host generalism.

We identify clearly distinct genotype clusters in generalist lineages inhabiting apparently identical niches (the same host animal) where homologous recombination with one another would theoretically prevent the emergence of distinct lineages. Genetic isolation of these clusters could not be explained by an inherent mechanistic barrier to recombination, because the strains freely recombined in the laboratory. Supported by evidence of marked differences in core and accessory genome variation, including genes associated with metabolic functions, we interpret these findings as indicating cryptic niche structure limiting opportunities for recombination of the two populations in nature. Our study offers a complementary approach to functional genome analyses, making use of the increased availability of large population genomic data sets to decipher the relationship with the ecology that gave rise to them.

## Materials and methods

### Bacterial culturing and genome sequencing

*Campylobacter jejuni* isolates were cultured and sequenced as described in the previous studies (Sheppard *et al.* 2013). Briefly, 128 isolates including multiple isolates from the ST-21 and ST-45 clonal complexes (Table S2, Supporting information) were subcultured and grown overnight in a microaerophilic workstation (5% CO_2_, 5% O_2_, 3% H_2_ and 87% N_2_) at 42 °C on Columbia blood agar (CBA) plates with 5% defibrinated horse blood (Oxoid, Basingstoke, UK). Single colonies were picked onto fresh CBA plates, and genomic DNA extraction was carried out using the QIAamp® DNA Mini Kit (Qiagen GmbH, Hilden, Germany). The DNA was resuspended in 100–200 μL of the elution buffer supplied and stored at −20 °C. An Illumina Genome Analyzer was used to sequence isolates with a multiplex sequencing approach involving 12 separately tagged libraries sequenced simultaneously in two lanes of an eight channel GAII flow cell. The standard Illumina Indexing protocol involved fragmentation of 2 μg genomic DNA by acoustic shearing to enrich for 200 bp fragments, A-tailing, adapter ligation and an overlap extension PCR using the Illumina 3 primer set to introduce specific tag sequences between the sequencing and flow cell binding sites of the Illumina adapter. DNA cleanup was carried out after each step to remove DNA <150 bp using a 1:1 ratio of AMPure® paramagnetic beads (Beckman Coulter, Inc., USA), and a qPCR was used for final DNA quantification. The average output was approximately 80 Mbp per isolate of high coverage short reads (25–50 bp). Contiguous sequences of 10–200 kb were assembled de novo using Velvet software (Zerbino & Birney 2008). Genome data that have been published elsewhere were archived in the Dryad repository (doi:10.5061/dryad.28n35).

### Phylogenetic analyses

A phylogeny of whole-genome alignments (1.53 Mbp) was reconstructed using MEGA version 3.1 with the Kimura 2-parameter model and Neighbour-joining clustering. To obtain the maximum-likelihood estimate of a phylogenetic tree which is not distorted by recombination events, we used FastTree (Price *et al.* 2009) on inferred nonrecombinant core genome segments based on the output of BratNextGen software (Marttinen *et al.* 2012), see Section Recombination analysis. To root the tree, *C. jejuni* sequences were aligned with 125 contigs available for a *Campylobacter coli* genome. For each of the 480 core genes, BratNextGen output was used to quantify what fraction of the gene had been affected by significant recombination, and the genes with more than 30% of the sites included in recombinant segments were excluded from the alignment process. The resulting 353 genes were locally aligned with each of the *C. coli* contigs using the consensus sequence over the 128 *C. jejuni* isolates. BLOSUM50 was used as the scoring matrix, and the gap opening and extension costs were set to 8 and 4, respectively. The resulting highest scoring alignment was extended by patching both sides of the aligned contig sequence with indels, so that the aligned contig sequence had the same length as the corresponding gene sequence. For 352 genes, the resulting fraction of the gene contained within the highest scoring contig was on average 99.09% (SD 1.53%), indicating that these genes were sufficiently accurately aligned with the *C. coli* genome. For all genes, we then excluded all the parts estimated to be significantly recombinant by BratNextGen and used the remaining 255 185 bp of the alignment as an input to FastTree to reconstruct the ML tree. FastTree was used with the default settings involving general time reversible model and the CAT model for rate heterogeneity.

### Recombination analysis

To estimate the amount of recombination in the core genome data and to obtain an appropriate recombination-free input for a subsequent phylogenetic analysis, we used the BratNextGen software (Marttinen *et al.* 2012). As the method requires the relative genomic positions of the variable sites, we mapped 480 core genes present in all isolates to the reference genome NCTC11168 (Parkhill *et al.* 2001). For 70 of the 480 genes, there is a small overlap (0.19–2.56% of the gene length) on the reference genome. Therefore, we adjusted accordingly the start and end positions of each gene sequentially with respect to the reference to obtain an input for BratNextGen where the genomic distances between variable sites are determined. The resulting input file had 37 129 SNPs for the 128 isolates. A total of 20 iterations of HMM parameter estimation were performed, and BratNextGen detected 5 groups of isolates with shared significant (*P*-value not exceeding 5%) recombinations over the core genome based 100 parallel permutation runs executed in parallel on a cluster computer. The negligible changes in HMM parameter values observed after half of the iterations indicated sufficient convergence in the estimation procedure. To establish genetically distinct groups of strains at genome-wide level, hierarchical BAPS clustering (Cheng *et al.* 2013) was performed using default settings with 10 clusters as the a priori upper bound. Exactly, identical clustering results were obtained in separate runs of the software.

### Host association of the major *C. jejuni* clonal complexes

Non-human host association information was extracted from the pubMLST database and broken down into three broad host categories: ‘chicken’, ‘cattle’ and ‘wild birds and environment’ corresponding to the source of isolation. A ternary plot was constructed using TriPlot version 4.1.2. An arbitrary cut-off for visual clarity was set up at 70–30% of prevalence for each group, to highlight specialist and generalist groups. A total of 2764 isolates were compared (ST-21 complex: 707 isolates; ST-45 complex: 549 isolates; ST-61 complex: 213 isolates; ST-257 complex: 209 isolates; ST-682 complex: 163 isolates; ST-42 complex: 147 isolates; ST-48 complex: 132 isolates; ST-177 complex: 110 isolates; ST-353 complex: 104 isolates; ST-206 complex: 91 isolates; ST-354 complex: 83 isolates; ST-443 complex: 64 isolates; ST-179 complex: 60 isolates; ST-573 complex: 43 isolates; ST-283 complex: 42 isolates; ST-661 complex: 36 isolates and ST-658 complex: 11 isolates).

### Core and accessory genome similarity

The core genome was defined as the group of genes present (not in a truncated variant) in all isolates and consisted of 595 individual loci. Orthologues at all these loci were defined in all isolates by comparison to the genome of isolate NCTC11168 (Parkhill *et al.* 2001). Reciprocal best hits to 11 168 loci, with at least 70% nucleotide identity and 50% difference in alignment length were obtained using the BLAST algorithm. The number of shared alleles between each combination of isolates was determined for isolates ordered using the whole-genome tree (see Section Phylogenetic analyses). The number of shared alleles between isolates varied from 4 to 593.

A list of accessory genes that were present in one but not all isolates was defined by aligning the *C. jejuni* genomes using progressiveMauve (Darling *et al.* 2008). Aligned segments <100 bp and those shared by all genomes (core) were removed and coding regions in the remaining noncore sequences (and possible gene functions) was determined using the Rapid Annotation using Subsystem Technology (RAST) server (Aziz *et al.* 2008). A list of 3485 accessory genes was identified. Reciprocal best hits using the BLAST algorithm were used to identify orthologues at all these loci as described for the core genome. Of 3485 accessory genes, 1128 genes were present in more than 5% and <95% of all strains. Patterns of gene presence and absence were investigated by constructing a binary matrix for these loci and calculating the number of shared genes at each accessory gene locus between each combination of isolates. The number of shared accessory genes varied from 282 to 1094. Matrices of gene presence or absence for all *C. jejuni* isolates were used to compare genes present either in ST-45 or ST-21 clonal complex isolates but not both.

### MLST population genetic analysis

We used a complete set of the available STs for *C. jejuni* from http://pubmlst.org/campylobacter/ as of the 26 May 2011. The database contained 3834 STs with sequences available for the following seven housekeeping genes: *aspA, glnA, gltA, glyA, pgm, tkt and uncA*. The MLST data were archived in the Dryad repository (doi:10.5061/dryad.28n35). BAPS software (Corander & Marttinen 2006) was used to cluster the sequences into genetically distinct groups and to estimate the level of admixture for each ST. The optimal clustering was obtained using 10 runs of the estimation algorithm with the prior upper bound of the number of clusters varying in the range [30, 100] over the 10 replicates. All estimation runs yielded highly congruent partitions of the ST data with exactly 23 clusters, indicating a strongly peaked posterior distribution in the neighbourhood of these partitions (estimated posterior probability 1.000). The admixture analysis was subsequently performed using the 23 clusters in the posterior model partition with 100 Monte Carlo replicates for allele frequencies and by generating 100 reference genotypes to calculate *P*-values. For reference cases, we used 10 iterations in estimation according to the user guidelines (Corander & Marttinen 2006). An ST was considered significantly admixed if the *P*-value did not exceed the threshold of 5%.

For each clonal complex (CC), we identified the BAPS cluster, which contained the largest fraction of STs from that particular CC. To quantify the levels of genetic connectivity among clonal complexes, we then calculated for each pair of CCs the relative amount of admixture with the main BAPS cluster for the other CC in the pair as the source, normalized by the number of STs in the CCs (Table S1, Supporting information). These values were then used to build a network for the CCs, such that each CC is represented by a node whose size is scaled with respect to the number of observed STs from cattle, chicken and wild bird/environmental sources in the study by Sheppard *et al*. (2011). The network was automatically created using the Force-Atlas algorithm in the open-source network visualization tool Gephi (v. 0.8.1) (Bastian *et al.* 2009). Each link between two nodes is represented by a weighted edge corresponding to the calculated relative genetic connectivity. Pairs of nodes for which the edge weight was smaller than 0.005 were defined as nonadjacent.

To assess how atypical the observed level of relative genetic connectivity between multihost ST-21 and ST-45 complexes was (0.0037, Table S1, Supporting information) compared with the overall distribution of such connectivity, we calculated the probability of observing at most as many admixed STs between ST-21 and ST-45 complexes as identified by BAPS, assuming that significantly admixed STs are uniformly distributed among groups. The resulting *P*-value was equal to 1.2144*10^−63^, which was obtained using cumulative binomial probability for a total of 707 cases consisting of 463 and 244 STs for the CCs ST-21 and ST-45, respectively, and 26 significantly (5% level) admixed STs between the two complexes.

### Transformation experiments

Transformation experiments were performed using isolates from the major multihost clonal complexes (ST-21 and ST-45 complexes) and a host-restricted lineage (ST-22 complex), with NCTC 11168 (CC ST-21) and 81–176 (CC ST-42) used as controls (Gaasbeek *et al.* 2009) (Table1). We followed previously published procedures (Wang & Taylor 1990; Gaasbeek *et al.* 2009) with ciprofloxacin resistance used as marker. Donor strains were grown on Nutrient agar (CM0271; Oxoid, Thermoscientific Oy, Finland) supplemented with 5% horse blood, hereafter referred to as Nutrient Blood Agar (NBA), at 42 °C for 24 h under microaerobic conditions (6.0% O_2_, 83.3% N_2_, 7.1% CO_2_ and 3.6% H_2_). The donor strains were made resistant to ciprofloxacin by serial passages on Müller–Hinton (MH) agar (CM0337; Oxoid, Thermoscientific Oy) supplemented with 5% horse blood and increasing ciprofloxacin concentrations, starting from 0.0625 μg/mL to 1 μg/mL ciprofloxacin. Ciprofloxacin resistance was confirmed by testing the MIC values [8 μg/mL for all donor strains, except 4791_2 cip (2 μg/mL)], and amplification and sequencing of the quinolone resistance-determining region of the *gyrA* gene (Griggs *et al.* 2005). Prior to the experiments, the median, minimum and maximum ciprofloxacin MICs for the recipient population were 0.030 μg/mL and (0.030–0.065) μg/mL, respectively. Transformation experiments were carried out in a biphasic cultivation medium containing NBA and Nutrient broth (CM0501; Oxoid, Thermoscientific Oy) (Gaasbeek *et al.* 2009). Recipient strains were grown on blood NBA at 37 °C for 16–18 h under microaerobic conditions. Bacterial recipient cells were harvested into 3 mL of Nutrient broth and the optical density at 600 nm (OD_600_) of the suspension was adjusted to 0.200 before transferring 500 μL to test tubes containing 2.50 mL slanted NBA. After 3 h at 37 °C under microaerobic conditions, 2.00 μg chromosomal DNA, isolated from *C. jejuni* donor strains, was added. Incubation was continued at 37 °C for 3 h under microaerobic conditions. The transformation frequency is given by the CFU/mL counted on NBA containing 1 μg/mL ciprofloxacin (NBAC) after substraction of spontaneous mutants, divided by the number of CFU/mL on nonselective NBA. The number of spontaneous mutants was determined by counting colonies on NBAC after incubation of recipient isolates for 6 h in the biphasic cultivation medium described above without adding donor DNA. The assumption that the number of colonies growing on NBAC was transformants after accounting for the spontaneous mutants was considered valid as the low ciprofloxacin MICs of the recipients precluded their growth on NBAC without donor DNA. The reported natural transformation frequencies were calculated for three biological replicates of transformant counts, each consisting of two technical replicates.

**Table 1:** Transformation experiment results ordered by most to least recombined

Direction of genetic exchange (Clonal complex to Clonal complex)	Donor		Recipient		Natural transformation frequency*
	ST	Isolate	ST	Isolate	
ST-21 to ST-45	ST-883	4791_2 cip	ST-137	277_1500	(6.0 ± 7.0) × 10^−6^
ST-45 to ST-21	ST-45	128_2 cip	ST-883	4791_2	(0.9 ± 1.6) × 10^−6^
ST-22 to ST-45	ST-22	22A cip	ST-137	277_1500	(1.4 ± 1.1) × 10^−6^
ST-22 to ST-45	ST-1947	FB 7095 cip	ST-137	277_1500	(1.1 ± 0.9) × 10^−6^
ST-45 to ST-21	ST-137	277_1500 cip	ST-883	4791_2	(9.7 ± 8.7) × 10^−7^
ST-21 to ST-22	ST-53	2403_3 cip	ST-22	FB 7143	(2.5 ± 2.5) × 10^−7^
ST-22 to ST-21	ST-1947	FB 7095 cip	ST-50	T-72455	(2.5 ± 1.9) × 10^−7^
ST-22 to ST-21	ST-22	22A cip	ST-53	2773	(2.9 ± 1.2) × 10^−7^
ST-21 to ST-22	ST-53	2773 cip	ST-22	22A	(2.1 ± 1.1) × 10^−7^
ST-45 to ST-21	ST-45	128_2 cip	ST-50	T-72455	(1.1 ± 0.8) × 10^−7^
ST-45 to ST-22	ST-45	128_2 cip	ST-22	FB 6329	(4.0 ± 4.8) × 10^−8^
ST-45 to ST-22	ST-45	4441 cip	ST-22	FB 6329	(6.0 ± 4.3) × 10^−8^
ST-45 to ST-22	ST-45	4441 cip	ST-22	FB 7143	(4.9 ± 3.5) × 10^−8^
ST-45 to ST-22	ST-45	128_2 cip	ST-22	FB 7143	(4.1 ± 3.3) × 10^−8^
ST-45 to ST-22	ST-45	4441 cip	ST-22	FB 6170	(3.5 ± 2.7) × 10^−8^
ST-22 to ST-45	ST-1947	FB 7095 cip	ST-45	70 316	(4.4 ± 2.2) × 10^−8^
ST-21 to ST-45	ST-50	T-72455 cip	ST-45	128_2	(1.7 ± 2.0) × 10^−8^
ST-21 to ST-45	ST-50	186_2349 cip	ST-45	128_2	(1.8 ± 1.8) × 10^−8^
ST-45 to ST-22	ST-45	128_2 cip	ST-22	FB 6170	(2.3 ± 0.9) × 10^−8^
ST-21 to ST-45	ST-883	4791_2 cip	ST-45	128_2	(1.0 ± 1.0) × 10^−8^
ST-45 to ST-21	ST-45	128_2 cip	ST-50	186_2349	(1.6 ± 2.4) × 10^−9^
ST-21 to ST-22	ST-53	2403_3 cip	ST-1947	76 455	<1.0 × 10^−9^

* The natural transformation frequency was determined by dividing the transformant colony-forming units per millilitre (CFU/mL), counted on NBA containing 1 μg/mL ciprofloxacin by the number of CFU/mL counted on nonselective NBA. The natural transformation frequencies are the average of three experiments, performed in duplicate.

## Results

**Figure 1:**
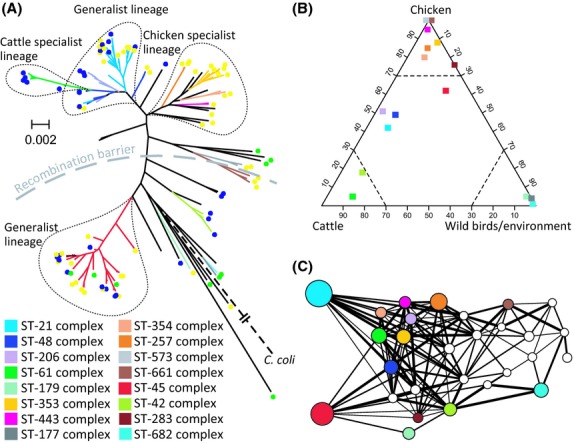
Genetic and ecological partitioning of *Campylobacter jejuni*. (A) Neighbour-joining tree of 128 *C. jejuni* genomes. Tree branches are coloured for the major multilocus sequence type (MLST) clonal complexes, and isolate source is indicated by coloured circles for samples from cattle (blue), chickens (yellow) and wild birds/environmental waters (green). The recombination barrier is based upon gene pool segregation derived using BRAT. The tree is rooted to *Campylobacter coli*, indicated by a broken black line, and the scale bar represents a genetic distance of 0.002. (B) Ternary plot of niche association of the major clonal complexes in the pubMLST database (pubMLST.org) for STs of 2764 isolates from cattle, chicken and wild bird/environmental sources. An arbitrary cut-off at 70–30% highlights specialist and generalist clonal complexes. (C) Network diagram where major clonal complexes (nodes) are scaled by the number of STs from cattle, chicken and wild bird/environmental sources in a published study (Sheppard *et al.* 2011). Lines link the nodes where there is admixture (>0.005) derived using BAPS. The thickness of admixture lines is proportional to the average amount of gene flow between STs (for each clonal complex) in four categories where average admixture is: 0.005–0.01; 0.011–0.04; 0.041–0.1; >0.1.

A phylogenetic tree of 128 isolate genomes (Table S2, Supporting information) estimated after removing recombinations showed that clustering was largely consistent with previously described clonal complex designations based on sharing of identical alleles at four or more MLST housekeeping gene loci (Feil *et al.* 2004) (Fig. 1A). The greater resolution provided by the whole-genome data also revealed common ancestry between some of these lineages, for example the ST-45 and ST-283 complexes clustered together, as did the ST-21, ST-48 and ST-206 complexes. Mapping isolate source information onto the tree provided evidence that some clonal complexes were associated with a single host and may have arisen from a common chicken or cattle associated ancestor (Fig. 1A). In contrast to this, isolates belonging to the ST-21 and ST-45 complexes – and the lineages that cluster with them – showed little evidence of historical separation by host, with isolates from cattle and chicken found at the end of several branches. A quantitative estimate of the degree of clonal complex host association was made by determining the number of times that 2764 STs of isolates from the PubMLST database (PubMLST.org) have been isolated from different host sources. This analysis revealed examples of specialist lineages together with others exhibiting generalist ecology that are frequently retrieved from multiple host species (Fig. 1B).

To investigate the nature of this genomic divergence, we conducted comparative analysis of the core and accessory genome. A total of 595 core gene loci, found in all isolates, were identified by BLAST comparison to the *Campylobacter jejuni* NCTC 11168 genome (Parkhill *et al.* 2001). In addition, 1128 accessory genes, not present in all isolates, were identified by aligning genomes with progressiveMauve (Darling *et al.* 2008) and assigning putative functions to noncore genes using the RAST server (Aziz *et al.* 2008). The number of shared core gene alleles and the similarity in accessory gene presence between each combination of isolates was broadly reflective of the phylogenetic relatedness (Fig. S1, Supporting information). The two major generalist groups, the ST-21/48/206 and ST-45/283 complexes, were distinct from one another with comparable patterns of genetic similarity in the core and accessory genome. Interestingly, the ST-61 complex, which clusters with the ST-21/48/206 complex, showed evidence for reduced genome similarity with these closely related lineages (Fig. S1, Supporting information). This could reflect reduced recombination between this cattle specialist and the generalist cluster from which it appears to have emerged.

Many of the genes that were mainly found in one generalist lineage and not the other were associated with particular metabolic functions (Table S3, Supporting information). For example, the L-fucose metabolic cluster (11 genes) was present in ST-21 complex and not in ST-45 complex isolates. L-fucose is an important host sugar found in the gut mucus and is a key fitness determinant for the *C. jejuni* reference strain NCTC 11168, from the ST-21 complex (Stahl *et al.* 2011; de Haan *et al.* 2012). In addition to this, differences in other accessory genes, such as those involved in iron and haem uptake, suggest adaptive differences between the two generalist lineages despite being found in the same hosts and a period of independent evolution.

To better understand the evolutionary history of separation of lineages, we estimated the number of ancestral *C. jejuni* populations by grouping isolates into genetically divergent clusters with shared MLST alleles using BAPS software (Corander & Marttinen 2006). Analysis of 3834 *C. jejuni* genotypes in the PubMLST database (Jolley & Maiden 2010) identified 23 genotype clusters, and these match well with the population structure described by the tree and MLST clonal complex designations (Fig. S2 and Table S4, Supporting information). The main advantage of this type of analysis is that it allows the identification of admixture events between clusters. *Campylobacter jejuni* is highly recombinogenic and this was confirmed by the network of homologous recombination between genotype clusters (Fig. 1C). The overall amount of admixture between ST-21 and ST-45 complexes and their interacting partners was not significantly higher when compared to other lineages (Table S1, Supporting information). However, consistent with their varied ecology, genotypes belonging to the generalist clonal complexes obtained DNA from more diverse sources, including host specialist lineages, reflecting their broader niches and increased opportunities for recombination. Strikingly, however, the host generalist ST-21 and ST-45 complexes, which contained 463 and 244 STs respectively, were found to be almost completely isolated from one another (*P*-value <0.0001), whereas the relative genetic connectivity was typically much higher between the generalist lineages and many of the specialists (Fig. 1C and Table S1, Supporting information).

A second analysis of admixture was conducted to investigate genome-wide patterns of recombination. Detailed analysis of the origin of recombining genes within the 128 genomes, using the Bayesian Recombination Tracker (BratNextGen) software confirmed that there were two broadly defined gene pools from which recombination events originated, one containing the ST-21 clonal complex and the other containing the ST-45 clonal complex with specialist lineages found in both (Fig. S3, Supporting information). Hierarchical BAPS clustering (Cheng *et al.* 2013) based on the genome sites not showing recombinant ancestry was highly congruent with the population division into separate generalist and specialist complexes and also reflected the earlier identified core genomic boundaries between and within the complexes (Fig. S1 and Table S5, Supporting information).

The absence of recombination between coexisting host generalists living in the same hosts is surprising as they appear to share identical ecological strategies being routinely isolated from the same cattle herd (Sproston *et al.* 2011), or chicken flock (Colles *et al.* 2011; Jorgensen *et al.* 2011) and therefore have ample opportunity for genetic exchange. One explanation is that they are mechanically or functionally incapable of exchanging DNA with one another. To test this hypothesis, we conducted in vitro transformation experiments, based on the assumption that in vitro recombination is related to mechanisms occurring in vivo (de Boer *et al.* 2002). Recipient strains from the major generalist clonal complexes (ST-21 and ST-45 complexes) and a host specialist complex (ST-22 complex) were grown in a biphasic cultivation medium containing DNA from ciprofloxacin resistant donor strains chosen from the same three clonal complexes. The results from these experiments showed that the natural transformation frequency (transformed CFU/mL divided by CFU/mL on nonselective media) varied among donor and recipient combinations from <1.0 × 10^−9^ to 1.1 × 10^−6^ CFU/mL. The number of spontaneous mutants was accounted for in the transformation frequency calculations and was on average 0.069 log CFU/mL, 95% CI [−0.233, 0.371]. Therefore, no evidence of an essential recombinational barrier was found, because genetic exchange occurred in both directions at a comparable rate between isolates from the ST-21, ST-45 and ST-22 complexes (Table1).

## Discussion

**Figure 2:**
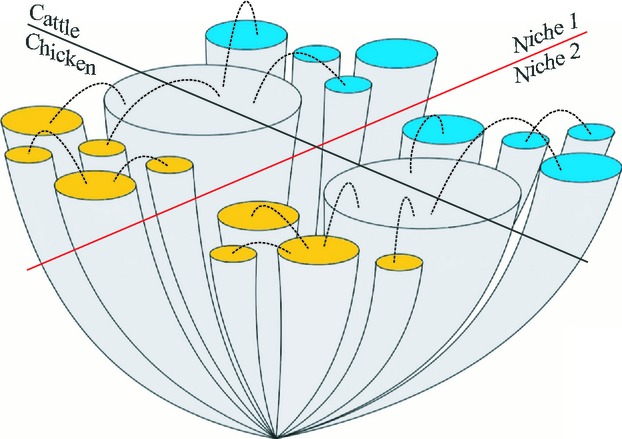
A scenario for the evolution of generalist *Campylobacter jejuni* lineages and a cryptic barrier to recombination. Lineages diverged from the common ancestor and clonal expansion led to the observed clonal complex structure. Some clonal complexes became associated with cattle (blue) and others with chicken (yellow) while two complexes were found in both hosts (grey) and can be considered generalists. Generalist complexes are able to recombine (broken lines) with specialist lineages across the host barrier (black diagonal line). However, the two generalists – and their recombinational partners – have separate gene pools and there is very little recombination between them (red diagonal line). This may indicate the presence of cryptic niche structure. The cross-sectional area and diameter of the lineage ‘trunks’ is roughly based on the abundance of isolates in this study, and the length of trunks is arbitrarily defined.

The breadth of ecological niches can vary among bacterial populations and lineages. Complex interplay of ecological factors can influence the development of highly stratified populations but the nature of the evolutionary forces acting at different levels is difficult to characterize. For example, in *Campylobacter jejuni,* the colonization of cattle and chickens has led to evolutionary specialization among some lineages that form host-associated genetic clusters. However, other lineages colonize multiple hosts but are influenced by other factors that are sufficient to impose a strong barrier to recombination (Fig. 2).

The simplest explanation for the coexistence of the lineages with different ecologies is that all *C. jejuni* lineages have the potential to colonize chicken and cattle hosts, but that the generalist lineages have relatively recently colonized them and have not yet had time to evolve host specialism. This hypothesis is not supported by the observation that generalist lineages have a comparable level of genetic diversity to specialist lineages and show no evidence of recent clonal expansion (Fig. 1A). In addition, there is evidence from in vivo infection experiments that suggests that different *C. jejuni* lineages have different host colonization potential (Waldenstrom *et al.* 2010). Both of these lines of evidence suggest an alternative explanation that the generalist lineages are genuinely better at colonizing multiple hosts and that this constitutes an independent ecological strategy. From the tree, it is clear that host generalism is not associated with a single lineage but with two phylogenetically divergent clusters, corresponding to the ST-21 and ST-45 clonal complexes. These lineages have divergent genetic backgrounds, possibly having emerged independently, but have convergent ecological strategies.

The observed genetic isolation between ST-21 and ST-45 clonal complexes cannot be explained simply by the homology dependence that favours recombination between similar DNA sequences (Cohan 2002; Fraser *et al.* 2007). First, because these lineages are exchanging DNA with other equally distantly related lineages. Second, because recombination occurred at a similar rate between genes at all levels of sequence divergence in spite of the mismatch repair mechanisms that can prevent the integration of sequences that contain even small numbers of nucleotide differences.

There are several possible scenarios that could interact to lead to the observed genetic isolation within *C. jejuni* populations. First, putative functional differences between lineages, inferred from accessory genome variation, suggest that these lineages may occupy different functional niches within the host. Consistent with this explanation, gut-associated bacteria in the same intestinal tract have been shown to occupy different microniches (Hayashi *et al.* 2005). Second, the resident microbiota might influence the colonization potential of different *C. jejuni* lineages and therefore the opportunity for genetic exchange. For example, colonization resistance (Buffie & Pamer 2013) could prevent the establishment of particular lineages and lead to predictable stratification of the population. Third, temporal variation in colonization could impede gene flow, for example through succession of different lineages (Lu *et al.* 2003). Additionally, some strains, termed residents, could colonize single individual hosts throughout increased periods during which other strains, termed transients, fail to establish as has been shown in gut dwelling *Escherichia coli* (Nowrouzian *et al.* 2005) and oral *Streptococcus mitis* (Hohwy *et al.* 2001) in humans. In *E. coli,* this temporal structuring has even been shown to vary between lineages (Nowrouzian *et al.* 2005).

There are undoubtedly other possible scenarios that lead to isolation among *C. jejuni* lineages and further work is necessary to investigate the nature of the cryptic niche structure. For example, to characterize the distribution of lineages among microniches, or interactions with the resident microbiota within the intestinal tract, *C. jejuni* from different locations could be isolated and cultured or examined *in situ* using techniques such as fluorescent labelling. This method has been used to demonstrate that different bacterial species occupy different locations in the chicken gut (Olsen *et al.* 2008). Additionally, temporal variation could be investigated through detailed quantification of succession of different lineages in controlled surveillance experiments to examine overlap, transient colonization and residency.

Whatever the nature of the cryptic niches, our results indicate that the observed genetic structure in *C. jejuni* populations is driven by multiple factors above and beyond the host species. Cryptic ecology within hosts may explain the invasion success of multiple coexisting *C. jejuni* lineages in domestic animals. A better understanding of this cryptic ecological niche structure may be important for understanding the emergence of zoonotic pathogens such as *Campylobacter* and determining the origin of human infection.

## Notes

J.C., S.K.S. and W.P.H. jointly designed the study and wrote the manuscript. J.C., G.M. and L.C. were primarily responsible for the genomic and statistical analyses, with some additional contributions from P.M. and T.R.C. A.K.L., C.P.A.H. and M.L.H. performed the transformation experiments. J.P. and S.D.B. sequenced the genomes and provided substantial contributions to interpretation of the data. A.V., A.R., F.C.H., N.J.C.S., K.F., F.M.C., K.A.J. and M.C.J.M. contributed to the acquisition of samples and study design. All authors carefully read and approved the final manuscript.

## Data accessibility

Whole-genome DNA sequences (sequence data for the 128 isolates); MLST gene DNA sequences, STs and non-human association information: Dryad: doi:10.5061/dryad.hk860.

## Supporting Information

Fig S1 Core and accessory genome variation in *Campylobacter jejuni*.

Fig S2 Relatedness and admixture among genotypes from the *Campylobacter* PubMLST database.

Fig S3 Analysis of the origin of recombining genes.

Fig S4 Homology dependence of recombination between *Campylobacter jejuni* clonal complexes

Table S1 Genetic connectivity of the clonal complexes measured by relative amount of significant admixture in BAPS analysis

Table S2 Isolate details.

Table S3 Function of genes found differentially in the ST-21 or ST-45 clonal complexes

Table S4 Source distribution of STs assigned to the 23 clusters detected by BAPS.

Table S5 Main clonal complex and hierarchical BAPS clusters based on genome-wide SNP data, excluding those sites that were significantly recombinant in the BratNextGen analysis.
