# Supplementary figures and images for: Cryptic ecology among host generalist Campylobacter jejuni in domestic animals
Source: Mol Ecol. 2014 Apr 25;23(10):2442–51. doi: 10.1111/mec.12742 (PMC4237157; doi:10.1111/mec.12742)

Core genome

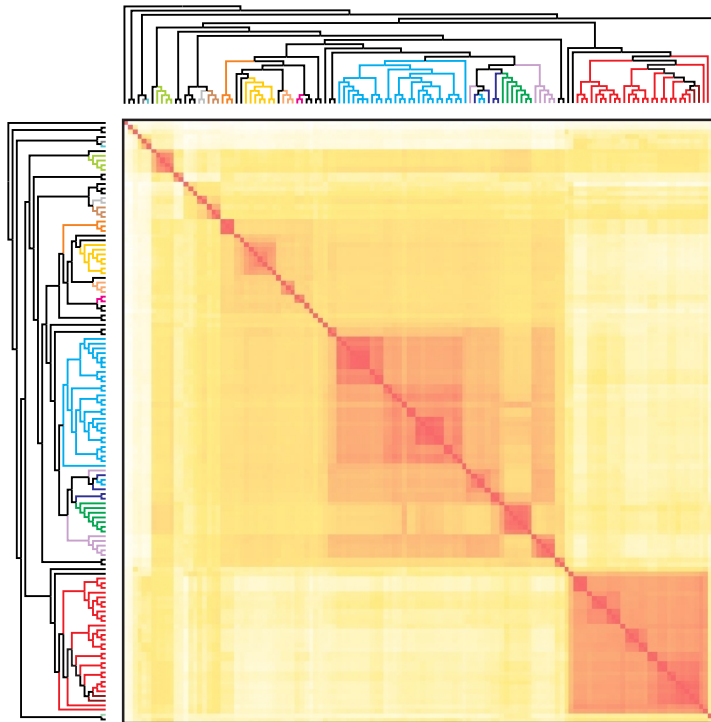

Accessory genome

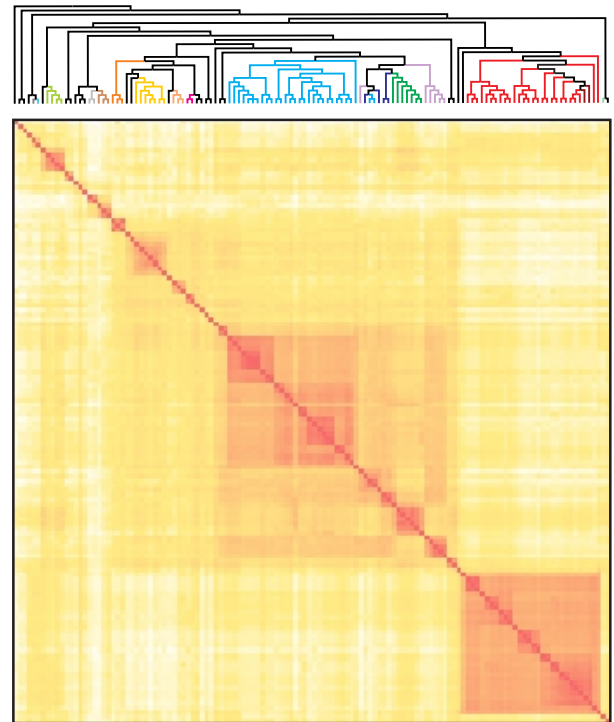

Supplement: Fig S1 — Core and accessory genome variation in Campylobacter jejuni. [file mec0023-2442-SD1.pdf]

A

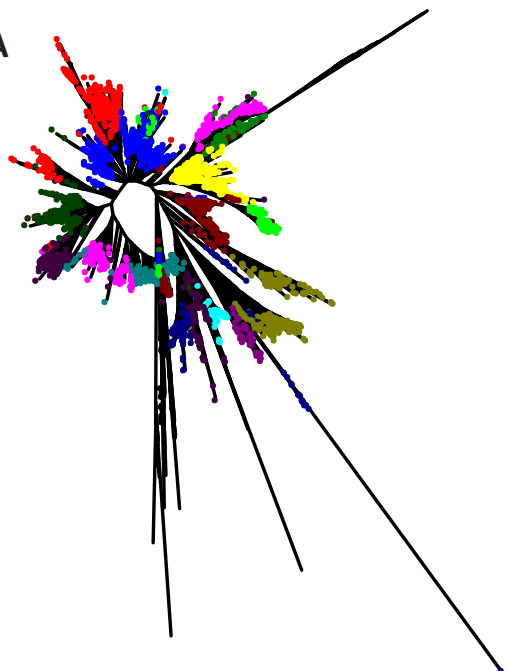

B

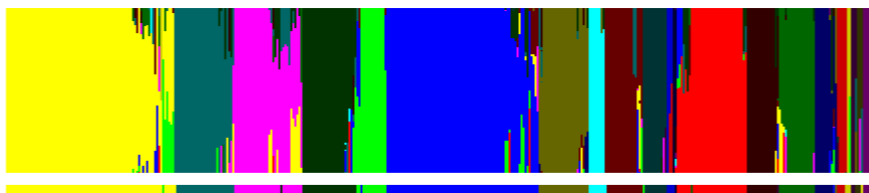

Supplement: Fig S2 — Relatedness and admixture among genotypes from the Campylobacter PubMLST database. [file mec0023-2442-SD3.pdf]

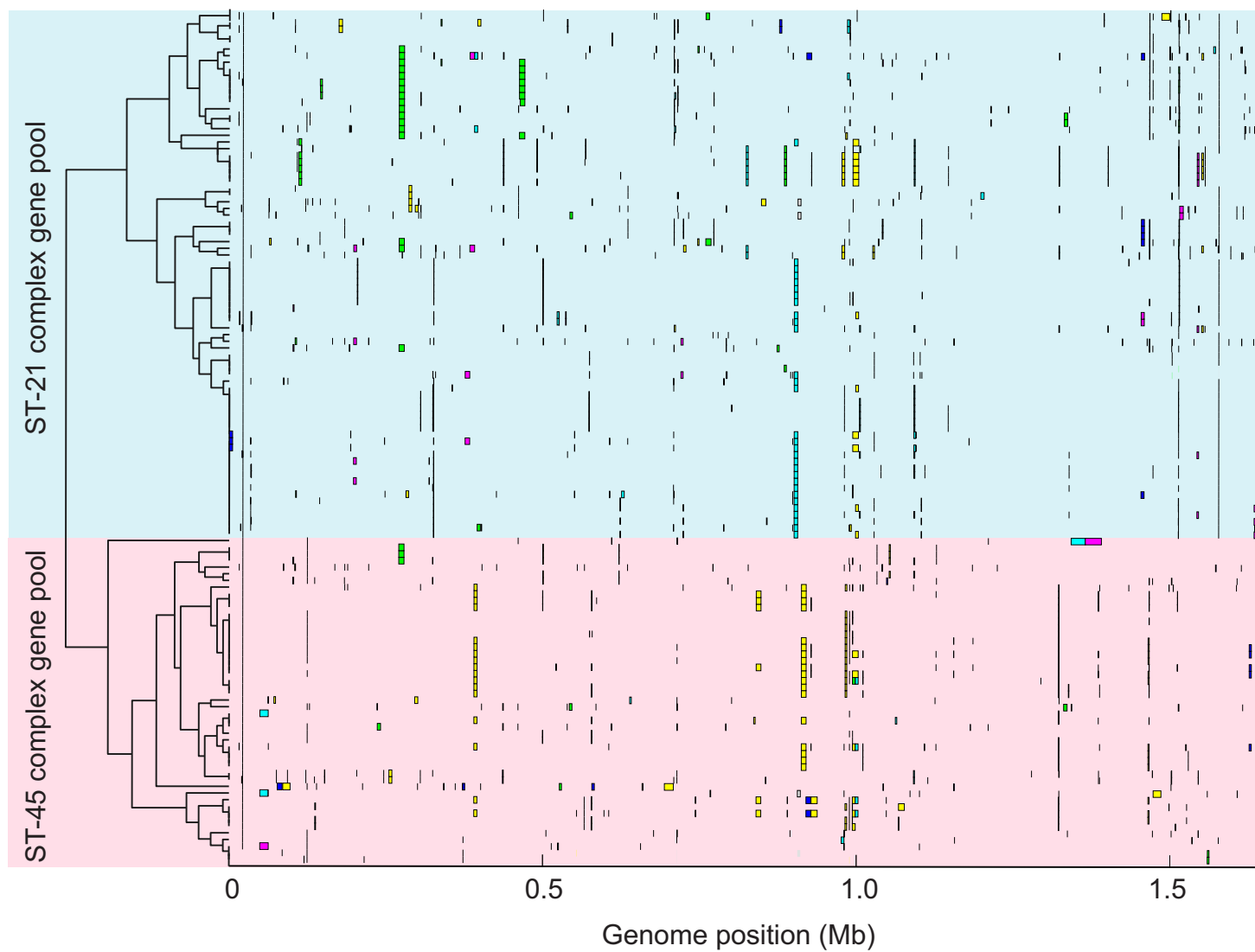

Supplement: Fig S3 — Analysis of the origin of recombining genes. [file mec0023-2442-SD5.pdf]

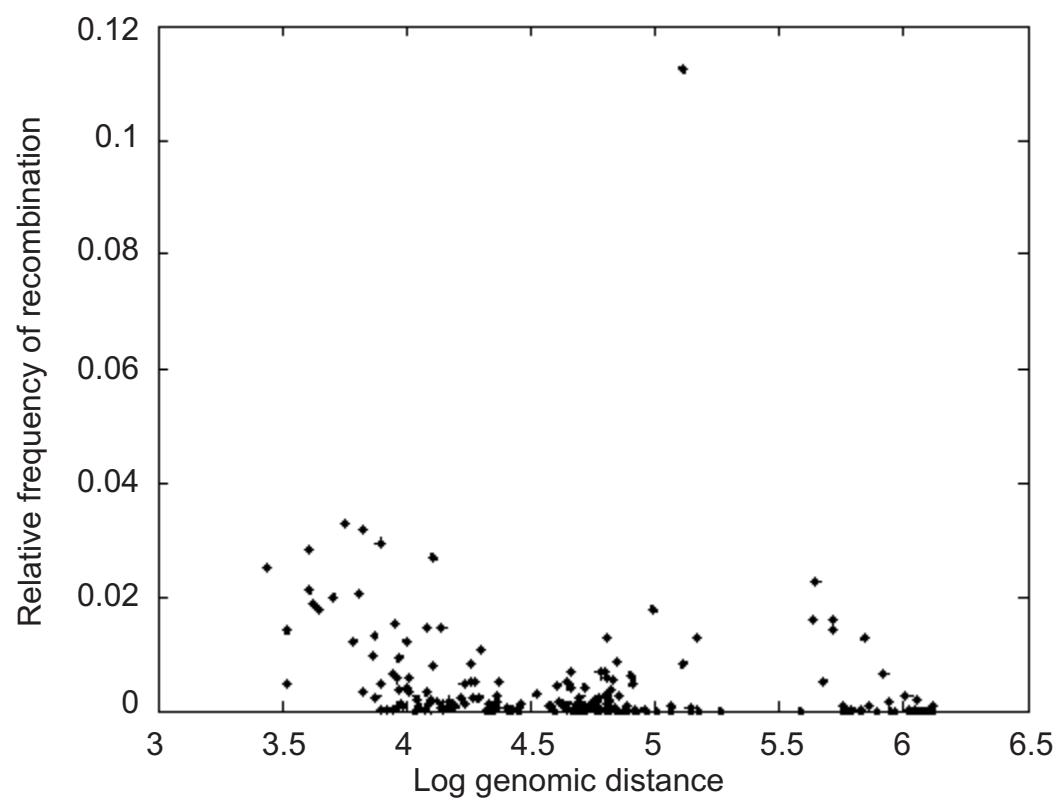

Supplement: Fig S4 — Homology dependence of recombination between Campylobacter jejuni clonal complexes [file mec0023-2442-SD7.pdf]
