# Supplementary material for: Cryptic ecology among host generalist Campylobacter jejuni in domestic animals
Source: Mol Ecol. 2014 Apr 25;23(10):2442–51. doi: 10.1111/mec.12742 (PMC4237157; doi:10.1111/mec.12742)
Supplement: Table S1 — Genetic connectivity of the clonal complexes measured by relative amount of significant admixture in BAPS analysis [file mec0023-2442-SD9.pdf]

Table S1 – Genetic connectivity of the clonal complexes measured by relative amount of significant admixture in BAPS analysis

	Clonal complex																											
	**21**	**22**	**42**	**45**	**48**	**52**	**61**	**177**	**179**	**206**	**257**	**283**	**353**	**354**	**403**	**443**	**446**	**508**	**573**	**574**	**658**	**661**	**677**	**682**	**702**	**1034**	**1275**	**1332**
**21**	0	0.047	0.047	0.004	0.009	0.117	0.005	0.001	0.001	0.011	0.004	0.004	0.011	0.011	0.047	0.011	0.117	0.001	0	0.117	0.047	0.004	0.001	0.001	0.004	0.004	0	0
**22**	0	0	0.066	0.022	0.010	0	0.009	0	0	0.007	0	0.022	0.007	0.007	0.066	0.007	0	0	0	0	0.066	0	0	0	0	0	0	0
**42**	0	0	0	0.126	0.008	0	0.024	0.005	0.005	0.002	0.005	0.126	0.002	0.002	0.318	0.002	0	0	0	0	0.318	0	0.005	0.005	0	0	0	0
**45**	0	0	0	0	0.020	0.011	0.007	0.005	0.008	0	0.002	0.211	0	0	0.013	0	0.011	0.002	0	0.011	0.013	0.002	0.008	0.005	0.002	0.002	0	0
**48**	0	0	0	0	0	0.003	0.003	0.004	0	0.069	0.004	0.014	0.069	0.069	0.043	0.069	0.003	0.001	0.003	0.003	0.043	0	0	0.004	0	0	0	0.003
**52**	0	0	0	0	0	0	0	0	0	0.007	0.006	0.003	0.007	0.007	0.057	0.007	0.097	0.003	0	0.097	0.057	0.002	0	0	0.002	0.002	0	0
**61**	0	0	0	0	0	0	0	0.001	0	0.138	0.002	0.002	0.138	0.138	0.006	0.138	0.005	0	0.003	0.005	0.006	0.004	0	0.001	0.004	0.004	0	0.003
**177**	0	0	0	0	0	0	0	0	0	0.003	0.025	0	0.003	0.003	0	0.003	0.002	0	0.008	0.002	0	0	0	0.123	0	0	0	0.008
**179**	0	0	0	0	0	0	0	0	0	0	0	0	0	0	0	0	0	0	0	0	0	0	0	0	0	0	0	0
**206**	0	0	0	0	0	0	0	0	0	0	0.011	0.003	0.130	0.130	0.007	0.130	0.047	0.003	0	0.047	0.007	0.001	0.001	0.002	0.001	0.001	0	0
**257**	0	0	0	0	0	0	0	0	0	0	0	0.007	0.061	0.061	0	0.061	0.034	0	0.002	0.034	0	0	0.001	0	0	0	0	0.002
**283**	0	0	0	0	0	0	0	0	0	0	0	0	0	0	0.015	0	0.008	0	0	0.008	0.015	0	0	0	0	0	0	0
**353**	0	0	0	0	0	0	0	0	0	0	0	0	0	0.025	0.016	0.025	0.017	0	0.001	0.017	0.016	0.001	0.003	0.001	0.001	0.001	0	0.001
**354**	0	0	0	0	0	0	0	0	0	0	0	0	0	0	0	0.125	0.001	0	0	0.001	0	0	0	0	0	0	0	0
**403**	0	0	0	0	0	0	0	0	0	0	0	0	0	0	0	0	0	0	0	0	0.013	0	0	0	0	0	0	0
**443**	0	0	0	0	0	0	0	0	0	0	0	0	0	0	0	0	0.052	0	0.003	0.052	0	0.003	0	0.002	0.003	0.003	0	0.003
**446**	0	0	0	0	0	0	0	0	0	0	0	0	0	0	0	0	0	0	0	0.239	0.002	0.011	0	0	0.011	0.011	0	0
**508**	0	0	0	0	0	0	0	0	0	0	0	0	0	0	0	0	0	0	0	0.015	0.006	0	0.179	0	0	0	0	0
**573**	0	0	0	0	0	0	0	0	0	0	0	0	0	0	0	0	0	0	0	0	0	0.019	0	0	0.019	0.019	0	0.441
**574**	0	0	0	0	0	0	0	0	0	0	0	0	0	0	0	0	0	0	0	0	0.007	0.023	0	0	0.023	0.023	0	0.002
**658**	0	0	0	0	0	0	0	0	0	0	0	0	0	0	0	0	0	0	0	0	0	0.010	0	0	0.010	0.010	0.002	0.001
**661**	0	0	0	0	0	0	0	0	0	0	0	0	0	0	0	0	0	0	0	0	0	0	0	0	0.316	0.316	0	0.041
**677**	0	0	0	0	0	0	0	0	0	0	0	0	0	0	0	0	0	0	0	0	0	0	0	0.027	0	0	0.001	0
**682**	0	0	0	0	0	0	0	0	0	0	0	0	0	0	0	0	0	0	0	0	0	0	0	0	0.002	0.002	0	0.006
**702**	0	0	0	0	0	0	0	0	0	0	0	0	0	0	0	0	0	0	0	0	0	0	0	0	0	0.050	0.001	0
**1034**	0	0	0	0	0	0	0	0	0	0	0	0	0	0	0	0	0	0	0	0	0	0	0	0	0	0	0	0.161
**1275**	0	0	0	0	0	0	0	0	0	0	0	0	0	0	0	0	0	0	0	0	0	0	0	0	0	0	0	0.006
**1332**	0	0	0	0	0	0	0	0	0	0	0	0	0	0	0	0	0	0	0	0	0	0	0	0	0	0	0	0
