# Supplementary material for: Cryptic ecology among host generalist Campylobacter jejuni in domestic animals
Source: Mol Ecol. 2014 Apr 25;23(10):2442–51. doi: 10.1111/mec.12742 (PMC4237157; doi:10.1111/mec.12742)
Supplement: Table S2 — Isolate details. [file mec0023-2442-SD10.pdf]

Table S2. Isolate details.

Isolate[1]	ID	Total length (bp)	source	country	year	ST[2]	clonal complex[3]
goose255	126	1663834	Goose	UK	2007	1033	1034
CjLMG23357	184	1694896	Clinical	Netherlands		4883	1275
goose3	129	1561449	Goose	UK	2007	696	1332
CT43850	122	1582720	Starling	UK	2007	177	177
CjLMG23211	214	1669921	Chicken	Belgium		220	179
CjLMG23264	179	1719111	Clinical	Slovenia		46	206
CjATCC43432	207	1703446	Clinical	Canada		122	206
7938-1	48	1671619	Cattle	UK	2006	206	206
130	99	1720953	Cattle	UK	2003	206	206
158	87	1715399	Cattle	UK	2003	273	206
Cj2008-988	192	1818384	Clinical	France		572	206
87	97	1629692	Cattle	UK	2003	19	21
7030A2	40	1726327	Chicken	UK	2006	21	21
8464	59	1705325	Cattle	UK	2006	21	21
8606	62	1658436	Cattle	UK	2006	21	21
7366	63	1679349	Chicken	UK	2006	21	21
185	89	1659711	Cattle	UK	2003	21	21
F12B1	110	1656261	Chicken	UK	2003	21	21
3	117	1697696	Clinical	UK	2003	21	21
NC_002163	29	1641481	Clinical	UK	2000	43	21
CjLMG9879	182	1650086	Clinical	Canada		47	21
Cj2008-1025	189	1656768	Clinical	France		50	21
Cj2008-831	195	1606170	Clinical	France		50	21
508-3754	71	1689713	Chicken	UK	2009	50	21
S07-0761	72	1692341	Chicken	UK	2009	50	21
508-2543	77	1692435	Chicken	UK	2009	50	21
508-2574	78	1693941	Chicken	UK	2009	50	21
Cj87330	203	1618859	Chicken	USA		50	21
274	60	1658292	Clinical	UK	2005	53	21
S07-2406	73	1651079	Chicken	UK	2009	53	21
10040	65	1761202	Chicken	UK	2006	104	21
195	94	1762939	Cattle	UK	2003	104	21
526	34	1643032	Clinical	UK	2005	262	21
S07-1597	74	1606379	Chicken	UK	2009	262	21
2850	36	1695272	Clinical	UK	2006	266	21
S08-0575	75	1693845	Chicken	UK	2009	266	21
Cj1928	211	1744799	Cattle	USA		806	21
5075	37	1667560	Clinical	UK	2006	883	21
F7B1	113	1665144	Chicken	UK	2004	883	21
Cj110-21	202	1615152	Cattle	USA		982	21

Table S2 - continued.

Isolate	ID	Total length (bp)	source	country	year	ST[1]	clonal complex[2]
Cj1997-11	199	1598427	Clinical	USA		22	22
F7A20	105	1697013	Chicken	UK	2004	257	257
48321	22	1573736	Chicken	UK	2001	257	257
S07-0533	80	1734017	Chicken	UK	2009	2030	257
NC 009839	26	1628115	Clinical	UK	2007	267	283
6760	54	1591217	Chicken	UK	2005	267	283
S07-4547	66	1776210	Chicken	UK	2009	353	353
Cj87459	204	1772922	Chicken	USA		452	353
Cj51037	201	1747053	Chicken	USA		939	353
Cj51494	173	1799590	Chicken	USA		4834	353
CjLMG23269	180	1735582	Chicken	Belgium		4837	353
Cj53161	187	1730741	Chicken	USA		4838	353
Cj1997-14	200	1767166	Clinical	USA		5159	353
508-2669	67	1688706	Chicken	UK	2009	354	354
508-2836	83	1667030	Chicken	UK	2009	354	354
NC 003912	28	1777831	Chicken	USA	2002	354	354
111	88	1716038	Cattle	UK	2003	270	403
CjATCC33560T	185	1714676	Cattle	Belgium		403	403
7620	42	1672737	Cattle	UK	2006	42	42
Cj129-258	172	1643841	Cattle	USA		459	42
NC 008787	27	1616554	Clinical	USA	2007	604	42
97	85	1654563	Cattle	UK	2003	3583	42
F2B7	106	1714044	Chicken	UK	2005	51	443
CjLMG9217	188	1663474	Clinical	Belgium		443	443
CjLMG23263	177	1739638	Chicken	Bosnia		3504	446
45	32	1650105	Clinical	UK	2005	11	45
508-2327	79	1645238	Chicken	UK	2009	11	45
C8	4	1596969	Chicken	UK	2005	45	45
7816	56	1607778	Cattle	UK	2006	45	45
508-2426	70	1595762	Chicken	UK	2009	45	45
S07-S237	82	1631119	Chicken	UK	2009	45	45
508-2944	84	1605483	Chicken	UK	2009	45	45
113	90	1603131	Cattle	UK	2003	45	45
218	91	1602224	Cattle	UK	2003	45	45
222	92	1607691	Cattle	UK	2003	45	45
234	104	1654338	Cattle	UK	2003	45	45
F4A6	111	1649834	Chicken	UK	2004	45	45
F7C3	112	1618948	Chicken	UK	2004	45	45
5	119	1621668	Clinical	UK	2003	45	45
CT86857_2.6.4	124	1603669	Starling	UK	2007	45	45
Duck184	131	1616162	Duck	UK	2007	45	45

Table S2 - continued.

Isolate	ID	Total length (bp)	source	country	year	ST[1]	clonal complex[2]
Cj55037	181	1598300	Chicken	USA		45	45
124	100	1626334	Cattle	UK	2003	137	45
goose95	128	1600143	Goose	UK	2007	137	45
4153	55	1625711	Clinical	UK	2006	230	45
F9C2	114	1633592	Chicken	UK	2004	230	45
8697	52	1616567	Cattle	UK	2006	334	45
6764	57	1620325	Chicken	UK	2005	334	45
39	103	1641790	Cattle	UK	2003	334	45
7648-2	45	1609163	Chicken	UK	2006	583	45
165	102	1607515	Cattle	UK	2003	583	45
S07-0175	81	1617200	Chicken	UK	2009	1003	45
6618B	39	1616482	Chicken	UK	2005	2219	45
8058	49	1663148	Cattle	UK	2006	38	48
Cj1893	210	1700567	Cattle	USA		38	48
CjLMG23218	175	1677541	Chicken	Belgium		48	48
Cj1997-4	196	1669690	Clinical	USA		475	48
Cj86605	183	1637051	Chicken	USA		4840	48
Cj1213	206	1673339	Cattle	USA		132	508
CjLMG9081	186	1593688	Clinical	SouthAmerica		52	52
508-3501	68	1838022	Chicken	UK	2009	573	573
F6_8	107	1838061	Chicken	UK	2004	1079	573
F10A2	108	1743461	Chicken	UK	2004	574	574
Cj2008-872	191	1605881	Clinical	France		61	61
7802A	13	1551351	Cattle	UK	2006	61	61
82	86	1659558	Cattle	UK	2003	61	61
Cj1997-7	197	1633444	Clinical	USA		61	61
Cj1798	208	1604776	Cattle	USA		61	61
Cj140-16	205	1677874	Cattle	USA		5161	61
Cj1997-1	193	1603628	Clinical	USA		658	658
7031	64	1698035	Chicken	UK	2006	814	661
F8C6	109	1759789	Chicken	UK	2004	814	661
508-3789	69	1821236	Chicken	UK	2009	2568	661
CjLMG9872	212	1619961	Clinical	Sweden		677	677
CL95044_4.6.4	125	1578916	Starling	UK		1020	682
Duck229	130	1669953	Duck	UK	2007	702	702
Cj23210	213	1762534	Chicken	Belgium		380	-
CjLMG23223	176	1701738	Chicken	Belgium		791	-
Cj1854	209	1619643	Cattle	USA		922	-
Cj2008-894	190	1627102	Clinical	France		1962	-
Cj2008-979	194	1798587	Clinical	France		2274	-
PH325	14	1543877	Environmental waters	New Zealand	2001	2381	-

Table S2 - continued.

Isolate	ID	Total length (bp)	source	country	year	ST[1]	clonal complex[2]
CjLMG23216	174	1708741	Chicken	Belgium		4835	-
Cj60004	178	1674182	Chicken	USA		4836	-
Cj1997-10	198	1791227	Clinical	USA		4839	-
7487	30	1613621	Clinical	UK	2007	-	-
77	96	1894874	Cattle	UK	2003	-	-
585	116	1656471	Clinical	UK	2005	-	-

[1]Isolates with a Cj prefix were published elsewhere.

[2]Sequence type (ST) was derived from the allelic profile of 7 housekeeping genes by multilocus sequence typing (MLST) and confirmed by whole genome sequencing.

[3]Clonal complexes are defined as including any ST that matches a previously defined central genotype (http://pubmlst.org/campylobacter/) at three or more loci.
