# Supplementary material for: Cryptic ecology among host generalist Campylobacter jejuni in domestic animals
Source: Mol Ecol. 2014 Apr 25;23(10):2442–51. doi: 10.1111/mec.12742 (PMC4237157; doi:10.1111/mec.12742)
Supplement: Table S3 — Function of genes found differentially in the ST-21 or ST-45 clonal complexes [file mec0023-2442-SD11.pdf]

Table S3 – Function of genes found differentially in the ST-21 or ST-45 clonal complexes

Present in ST-21 complex and absent from >90% ST-45 complex isolates		
**Gene[1]**	**Function**	**Ref**
Cj0055c	Unknown	(1)
Cj0056c	unknown	(1)
Cj0057	possible periplasmic protein	(1)
Cj0058	possible periplasmic protein	(1)
Cj0122	unknown	(1)
Cj0177	putative iron transport protein	(2)
Cj0178	TonB dependent receptor	(2,3)
exbB1	putative iron transport protein	(4)
exbD1	putative iron transport protein	(4)
tonB1	putative iron transport protein	(4)
Cj0380c	unknown	(1)
Cj0424	Functional link to iron stimulation	(4,5)
Cj0425	Functional link to iron stimulation	(4,5)
Cj0480c	L-fucose metabolism	(6)
dapA	L-fucose metabolism	(1)
uxaA'	L-fucose metabolism	(1)
uxaA'	L-fucose metabolism	(1)
Cj0484	L-fucose metabolism	(1)
Cj0485	L-fucose metabolism	(1)
Cj0486	L-fucose metabolism	(1)
Cj0487	L-fucose metabolism	(1)
Cj0488	L-fucose metabolism	(1)
ald'	L-fucose metabolism	(1)
ald'	L-fucose metabolism	(1)
Cj0566	unknown	(1)
Cj0567	putative iron transport protein	(4)
Cj0568	unknown	(1)
Cj0569	unknown	(7)
Cj0618	Possibly involved in flagellin glycosylation	(1)
Cj0690c	Type I restriction enzyme	(1)
Cj0737	Putative adhesin	(1)
Cj0738	unknown	(1)
Cj0739	unknown	(1)
Cj0740	unknown	(1)
Cj0747	unknown	(1)
Cj0748	unknown	(8)
tonB3	Iron regulated tonB protein	(4)
cfrA	putative iron uptake protein (ferric receptor)	(4)
Cj0818	putative iron- and Fur-regulated lipoprotein	(1)
Cj0819	putative iron transport protein	(4)
Cj1122c	unknown	(1)
Cj1135	lipooligosaccharide synthesis	(1)
Cj1297	antimicrobial efflux pump	(1)
hsdR	putative type I restriction enzyme protein	(1)
rloH	putative ATP/GTP-binding protein	(1)
hsdS	putative type I restriction enzyme protein	(1)
mloB	unknown	(1)
hsdM	putative type I restriction enzyme protein	(1)

Cj1555c	hypothetical protein	(1)
Cj1585c	putative oxidoreductase	(1)
Cj1668c	putative periplasmic protein	(1)
Cj1725	putative periplasmic protein	(1)
metA	homoserine O-succinyltransferase	(5)
metB	putative O-acetylhomoserine (thiol)-lyase	(5)
PAN0484	hypothetical protein	-
PAN0513	hypothetical protein	-
PAN1954	hypothetical protein	-
PAN2855	Predicted metal-dependent hydrolase	-
PAN2856	putative tartrate transporter	-
PAN2879	putative periplasmic protein	-
PAN3003	unknown	-
PAN3004	unknown	-
PAN3069	hypothetical protein	-
PAN3181	hypothetical protein	-
PAN3324	putative periplasmic protein	-
PAN3325	putative periplasmic protein	-

Present in ST-45 complex and absent from >90% ST-21 complex isolates

**Gene**[1]	**Function**	**Ref**
PAN0180	3',5'-cyclic-nucleotide phosphodiesterase	-
PAN0367	4-carboxymuconolactone decarboxylase	-
PAN1906	alginate O- acetylation protein	-
PAN0370	Anaerobic dimethyl sulfoxide reductase chain A	-
PAN2385	Arsenic efflux pump protein	-
PAN1864	arylsulfate sulfotransferase	-
PAN0958	binding-protein-dependent transport system component	-
PAN1841	Bll0374 protein	-
PAN1696	conserved hypothetical protein	-
PAN1302	DNA methylase-type I restriction-modification system	-
PAN1761	DNA methylase-type I restriction-modification system	-
PAN1061	Ferrochelatase, protoheme ferro-lyase	-
PAN0137	hypothetical protein	-
PAN0387	hypothetical protein	-
PAN2369	hypothetical protein	-
PAN0368	hypothetical protein	-
PAN0390	hypothetical protein	-
PAN0361	hypothetical protein	-
PAN0384	hypothetical protein	-
PAN2592	hypothetical protein	-
PAN0953	hypothetical protein	-
PAN1543	hypothetical protein	-
PAN0363	hypothetical protein	-
PAN1297	hypothetical protein	-
PAN0608	hypothetical protein	-
PAN1851	hypothetical protein	-
PAN1006	hypothetical protein	-
PAN3380	hypothetical protein	-
PAN0358	Glucose-1-phosphate thymidylyltransferase	-
PAN2754	Hemerythrin-like iron-binding protein	-
PAN0035	hypothetical protein	-

PAN0037	hypothetical protein	-
PAN0364	hypothetical protein	-
PAN0385	hypothetical protein	-
PAN0386	hypothetical protein	-
PAN0594	hypothetical protein	-
PAN0746	hypothetical protein	-
PAN0957	hypothetical protein	-
PAN1490	hypothetical protein	-
PAN1491	hypothetical protein	-
PAN1535	hypothetical protein	-
PAN1616	hypothetical protein	-
PAN1793	hypothetical protein	-
PAN1849	hypothetical protein	-
PAN2215	hypothetical protein	-
PAN2216	hypothetical protein	-
PAN0388	IncF plasmid conjugative transfer protein TraG	-
PAN3295	IncF plasmid conjugative transfer protein TraG	-
PAN0389	IncF plasmid conjugative transfer protein TraN	-
PAN0374	lipopolysaccharide core biosynthesis protein LpsA	-
PAN0609	MGC82361 protein	-
PAN0359	Probable poly(beta-D-mannuronate) O-acetylase	-
PAN1630	Putative periplasmic protein	-
PAN1663	Putative periplasmic protein	-
PAN0391	Putative phage protein	-
PAN2285	RloB	-
PAN1536	sugar ABC transporter, periplasmic sugar-binding protein	-
PAN0036	sugar binding protein of ABC transporter	-
PAN1441	TraG-like protein	-
PAN3202	transporter, putative	-
PAN3269	Type I restriction-modification enzyme M subunit	-
PAN0366	Type I restriction-modification system, DNA-methyltransferase	-
PAN0362	Type I restriction-modification system, restriction subunit R	-
PAN0365	Type I restriction-modification system, specificity subunit S	-
PAN2410	Type I restriction-modification system, specificity subunit S	-
PAN3397	Type I restriction-modification system, specificity subunit S	-
PAN0360	unknown	-

[1]Genes with a PAN prefix were identified in this study by aligning genomes with progressiveMauve (9) and assigning putative functions to non-core open reading frames using the RAST server (*10*).

References

1. J. Parkhill *et al.*, *Nature* **403**, 665 (2000).
2. C. E. Miller, P. H. Williams, J. M. Ketley, *Microbiology* **155**, 3157 (Oct, 2009).
3. K. Palyada, D. Threadgill, A. Stintzi, *J Bacteriol* **186**, 4714 (Jul, 2004).
4. K. Holmes *et al.*, *Microbiology* **151**, 243 (Jan, 2005).
5. R. R. Chaudhuri *et al.*, *Microbiology* **157**, 2922 (Oct, 2011).
6. M. A. Javed *et al.*, *Microbiology* **156**, 1134 (Apr, 2010).
7. N. Dorrell *et al.*, *Genome Res* **11**, 1706 (2001).

8. A. Metris, M. Reuter, D. J. Gaskin, J. Baranyi, A. H. van Vliet, *BMC Genomics* **12**, 535 (2011).
9. A. E. Darling, I. Miklos, M. A. Ragan, *PLoS Genet.* **4**, e1000128 (Jul, 2008).
10. R. K. Aziz *et al.*, *BMC Genomics* **9**, 75 (2008).
