# Supplementary material for: Cryptic ecology among host generalist Campylobacter jejuni in domestic animals
Source: Mol Ecol. 2014 Apr 25;23(10):2442–51. doi: 10.1111/mec.12742 (PMC4237157; doi:10.1111/mec.12742)
Supplement: Table S5 — Main clonal complex and hierarchical BAPS clusters based on genome-wide SNP data, excluding those sites that were significantly recombinant in the BratNextGen analysis. [file mec0023-2442-SD13.pdf]

Table S5 – Main clonal complex and hierarchical BAPS clusters based on genome-wide SNP data, excluding those sites that were significantly recombinant in the BratNextGen analysis.

Clonal Complex	BAPS cluster A	BAPS cluster B	Generalist/Specialist[1]
21	1	1.1, 1.2	Generalist
48	1	1.3	Generalist
206	1	1.3	Generalist
61	1	1.4	Specialist
353	2	2.1	Specialist
443	2	2.1	Specialist
354	2	2.1	Specialist
257	2	2.2	Specialist
179	3	3.1	Specialist
177	3	3.1	Specialist
573	3	3.2	Specialist
661	3	3.2	Specialist
45	4	4.1, 4.2, 4.3, 4.4	Generalist
283	4	4.4	Specialist
42	3	3.3	Specialist
682	3	3.1	Specialist

[1]Generalist and specialist definitions are derived from STs of 2,764 isolates from cattle, chicken and wild bird/environmental sources with a 70%-30% cut off as in Figure 1B.
