# Supplementary material for: Cryptic ecology among host generalist Campylobacter jejuni in domestic animals
Source: Mol Ecol. 2014 Apr 25;23(10):2442–51. doi: 10.1111/mec.12742 (PMC4237157; doi:10.1111/mec.12742)
Supplement: Table S4 — Source distribution of STs assigned to the 23 clusters detected by BAPS. [file mec0023-2442-SD12.pdf]

Table S4– Source distribution of STs assigned to the 23 clusters detected by BAPS.

Cattle	Chicken	Duck	Environment	Goose	Pig	Sheep	Starling	Turkey	Wild bird	Cluster size
2	67	37	9	16	0	0	0	28	51	318
4	19	0	0	0	0	4	1	3	2	131
62	104	0	7	2	0	18	2	6	2	649
55	108	2	4	1	0	27	3	9	4	715
47	34	0	5	1	12	7	4	1	3	317
0	1	2	5	0	0	0	42	0	11	73
49	22	0	1	1	7	6	0	1	2	193
0	28	15	19	33	0	0	1	3	18	166
1	5	1	31	1	0	0	1	0	33	93
7	22	4	43	0	0	8	16	0	43	223
0	0	0	5	0	0	0	0	0	40	45
13	33	0	15	4	1	3	5	2	10	225
5	11	3	51	5	0	1	5	0	16	172
13	33	0	5	0	1	0	0	10	0	225
0	0	0	1	0	0	0	0	0	2	15
4	3	0	1	0	0	0	0	0	0	32
2	6	0	2	0	0	0	0	0	0	32
43	4	0	5	0	1	6	0	0	0	100
5	10	0	4	0	5	0	0	0	0	38
0	0	0	1	0	0	0	0	0	0	1
7	2	1	1	0	1	2	0	0	0	37
4	5	0	0	0	0	2	0	0	0	32
0	1	0	0	0	0	0	0	0	0	2
