# Supplementary material for: Cryptic ecology among host generalist Campylobacter jejuni in domestic animals
Source: Mol Ecol. 2014 Apr 25;23(10):2442–51. doi: 10.1111/mec.12742 (PMC4237157; doi:10.1111/mec.12742)
Supplement: Supplementary file 14 — Supplementary [file mec0023-2442-SD14.pdf]

Figure S1. Core and accessory genome variation in *C. jejuni*. Matrices show pairwise comparison between 128 isolates ordered according to phylogenetic tree. (A) Core genome similarity is based upon the number of shared alleles at 595 loci found in all isolates. (B) Accessory genome similarity based upon gene presence or absence at 1128 non-core loci. The heat-map colouring ranges from white, through yellow and orange, to red (maximum). The minimum number of shared alleles in the core genome is 4 and the maximum is 595. The minimum number of shared accessory genes is 282 and the maximum is 1,094.

Figure S2. Relatedness and admixture among genotypes from the *Campylobacter* PubMLST database. (A) Neighbour joining tree of the 3834 *C. jejuni* genotypes (concatenated MLST alleles) constructed by using MEGA5 and the Kimura two-parameter method. Individual genotypes are coloured by BAPS group. (B) Admixture between the 23 BAPS clusters. Each column represents a single multilocus sequence type, coloured according to the proportion of genetic variation assigned to each cluster. The final cluster assignment is shown by the color of the line underneath.

Figure S3. Analysis of the origin of recombining genes. Recombination events inferred from BRAT analysis of 128 genomes. The tree was constructed based upon the similarity of recombined sequence. Based on this there are two recombinational gene pools, one containing the ST-21 complex lineages (blue) and the other containing those from the ST-45 complex (red) with specialist lineages found in both.

Figure S4. Homology dependence of recombination between *C. jejuni* clonal complexes. The relative frequency of recombination at different levels of nucleotide divergence. Recombination is slightly more common between less divergent sequences but also occurs at areas of the genome where there is high divergence between genes. There is no clear trend indicating that recombination is homology dependent.
